# The Preparation and Properties of Porous Sepiolite Ceramics

**DOI:** 10.1038/s41598-019-43918-9

**Published:** 2019-05-14

**Authors:** Li Tian, Lijuan Wang, Kailei Wang, Yuedan Zhang, Jinsheng Liang

**Affiliations:** 1 Key Laboratory of Special Functional Materials for Ecological Environment and Information (Hebei University of Technology), Ministry of Education, Tianjin, 300130 People’s Republic of China; 2 Institute of Power Source and Ecomaterials Science, Hebei University of Technology, Tianjin, 300130 People’s Republic of China

**Keywords:** Structural properties, Ceramics

## Abstract

In this paper, a new type of porous ceramics was prepared using the raw sepiolite mineral. The porous ceramics was shaped by the dry pressing method and sintered in the range of 700 ~ 1200 °C. The temperature-microstructure evolution and the properties of porous sepiolite ceramics were investigated by thermo gravimetric and differential thermal analyses (TG-DTA), X-ray diffraction (XRD), bending strength, compressive strength, scanning electron microscopy (SEM) and mercury intrusion porosimeter (MIP). The sintering kinetics of the porous ceramics from sepiolite was investigated by means of stepwise isothermal dilatometry (SID). The mechanical properties improved with the increasing sintering temperature, and the bending strength and compression strength reached a maximum of 52 MPa and 32 MPa respectively at 1200 °C. The porosity increased with the sintering temperature until 1100 °C attaining the value of 55.40% and then decreased to a value of 46.48% at 1200 °C. The main crystal phases of the porous ceramics were akermanite and diopside. At 1200 °C, the pores inside the ceramics basically follows a unimodal distribution, which was mainly located near 553 nm. The sintering activation energy of porous sepiolite ceramics was measured by step isothermal thermal expansion with a value of 791.42 kJ/mol in the range of 1000 °C to 1200 °C.

## Introduction

For the theoretical high surface area, excellent permeability, good chemical and thermal stability, low electrical and thermal conductivity^1–5^, porous ceramics are widely used in chemical, energy, metallurgy, bio-medicine, environmental protection, aerospace and other fields^6–10^, as filter materials, catalyst supports, insulation materials, bio-functional materials and so on. Generally a porous material is needed. The pore forming methods for the preparation of suitable porous ceramics include volatiles addition method, sol-gel method^11^, freeze drying method^12^, bubble method^13^ and a proper sintering conditions control method. However, due to the high cost, environmental pollution and processing difficulty, the wide application of porous ceramics is sometimes restricted.

In recent years, there are increasing concerns about the raw clay mineral materials used to prepare porous ceramics, which are abundant, cheap, environment-friendly and have a low sintering temperature. A macroporous ceramic support was fabricated using inexpensive clays available in India by uniaxial compaction technique^14^. Sepiolite is a natural hydrated magnesium silicate clay mineral with a micro fibrous morphology and good sorptive property. Brauner and Preisinger^15^ deduced the structure of the sepiolite by using the X-ray diffraction method as the orthorhombic system with space group Pncn. Sepiolite belongs to the structural family of 2:1 phyllosilicates, which is a hydrous magnesium silicate with (Si_12_Mg_8_O_30_)(OH)_4_(OH_2_)_4_·8H_2_O as the theoretical unit cell formula^16,17^. The sepiolite includes a tubular channel, which section size is 0.37 × 1.06 nm^18^. The form of sepiolite clay are mostly fibrous or fiber bundle under the scanning electron microscopy^19,20^, although the length of sepiolite fibers varies greatly from different places^17^. The process of dehydration and dehydroxylation of sepiolite reported in many articles is generally considered to occur in three stages^21–24^. Most studies report that zeolite water is removed from room temperature up to 400 °C, the water of crystallization is removed in the 500 to 800 °C range, and finally hydroxyl groups in sepiolite are removed at around 800 °C and above.

Sepiolite is widely used in a variety of fields due to its special structure and the abundant sources in China (about 30% of the world’s reserves of this clay mineral) including cosmetics, ceramics, detergents, paper and paint^25–28^. The adsorption capacity of sepiolite makes it a valuable bleaching agent, purifying agent, filter and carrier^29,30^. The rheological properties can be used as thickening agent, suspension agent and as a thixotropic agent^31^. The acicular particles of sepiolite are easily interspersed into a network structure in water or a polar solvent to form a highly viscous suspension having better rheology. It improves the suspension properties, fluidity and plasticity of the mud. After the mud is dried, it becomes a solid, porous structure with almost no cleavage plane, which increases the strength of the ceramic body^32^. Yan *et al*.^33^ found that the addition of sepiolite improved the mechanical properties of bone china. The mechanism of toughening of sepiolite was fiber pull-out and weak interfacial effect. However, excessive addition of sepiolite leads to an increase in pore structure.

In this work, the raw sepiolite mineral that can be applied in future industry was used as a raw material to prepare a novel type of porous ceramics. The temperature-microstructure evolution and the properties of the sepiolite porous ceramics during the sintering processing were investigated. The sintering kinetics of the porous ceramics is well analyzed by means of stepwise isothermal dilatometry (SID) to obtain apparent activation energy.

## Materials and Methods

### Raw materials

**Figure 1:**
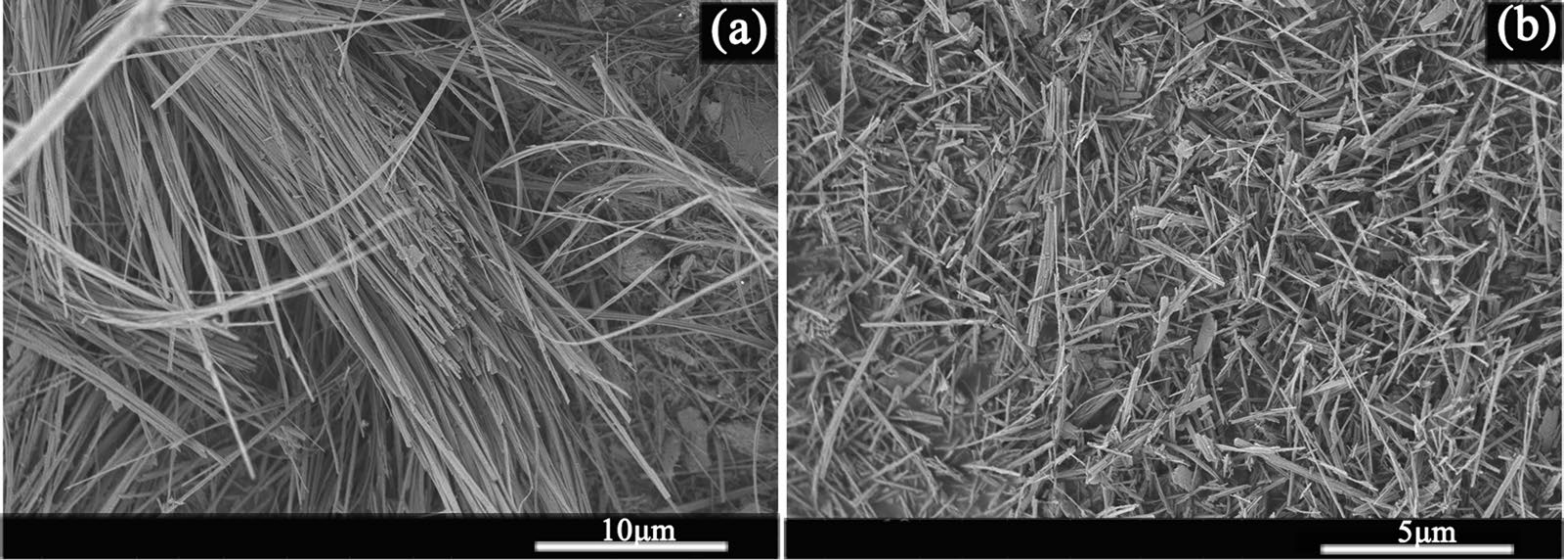
The SEM images of sepiolite (**a**) Raw sepiolite (**b**) Airflow milled sepiolite.

The raw sepiolite mineral used in the work was sourced from Henan province in China, with a density of 2.4 ~ 2.6 g/cm^3^. The chemical composition of the sepiolite is shown in Table 1 and the main chemical compositions were SiO_2_, MgO and CaO. The raw sepiolite mineral was pre-dispersed by the airflow mill and then ground by a ball mill for 20 min under the condition of 350 r/min with the ball-material ratio of 1:1. After that the resulting sepiolite powder was passed through a 60 mesh screen. As can be seen from Fig. 1, the airflow milled sepiolite has a looser structure and a shorter length of approximately 2 to 4 μm compared to the raw sepiolite, see the scanning electron microscopy image in Figure 1. A typical composition of the sepiolite from Henan is presented in Table 1 below.

**Table 1:** Chemical composition of Henan sepiolite.

Oxide	SiO_2_	CaO	MgO	Al_2_O_3_	Fe_2_O_3_	MnO	TiO_2_	K_2_O	Na_2_O	Total
Content/wt%	39.7	32.4	17.0	6.20	3.46	0.40	0.27	0.21	0.20	99.84

In addition to the sepiolite, another auxiliary material polyvinyl alcohol, was blended with the sepiolite powder to improve the agglomeration ability for well shaping. The polyvinyl alcohol was formulated as an aqueous solution with a concentration of 1 wt%. This concentration of polyvinyl alcohol aqueous solution was added to sepiolite in an amount of 5 wt%.

### Preparation of the samples

**Figure 2:**
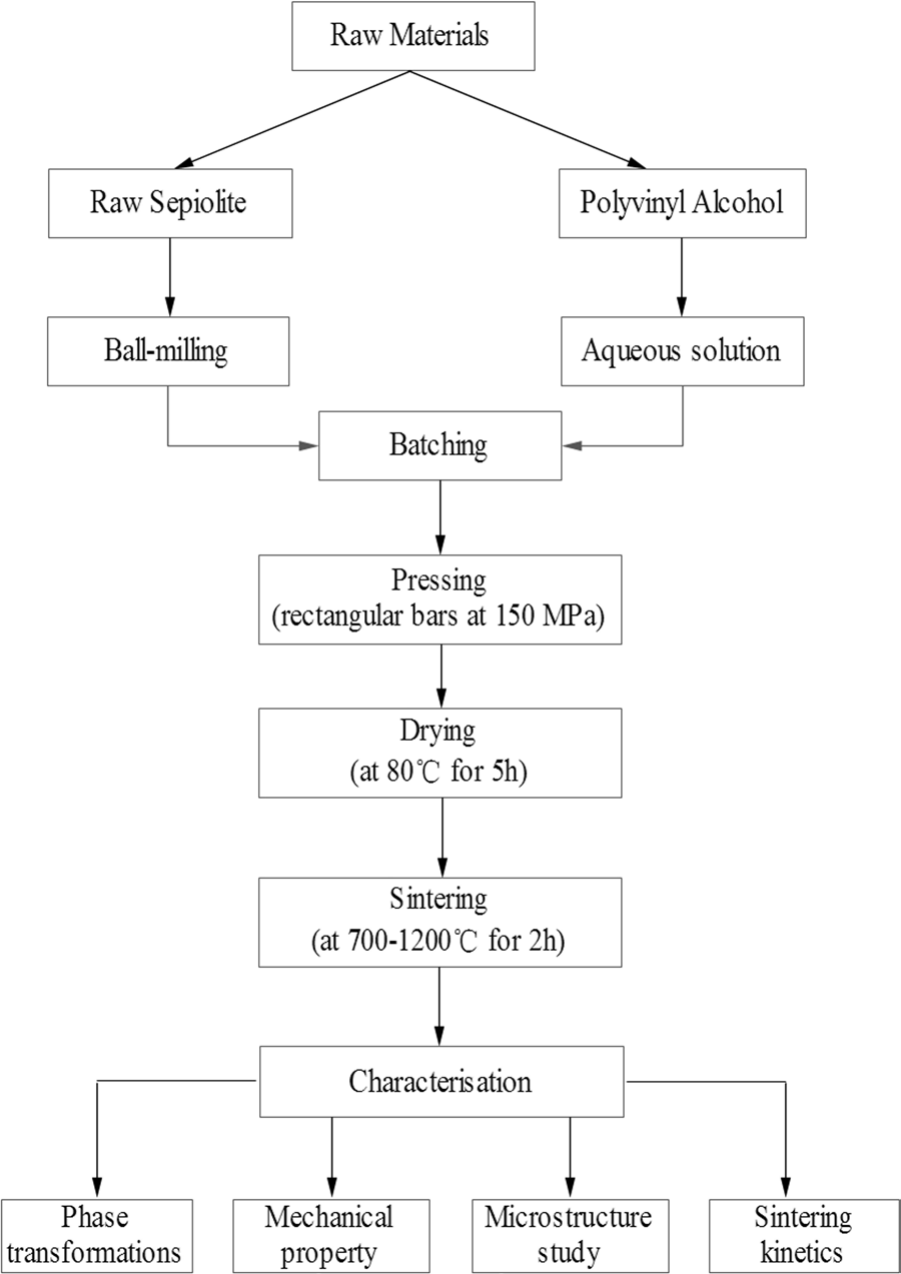
Flow chart for the processing and characterization of the porous ceramic made by raw sepiolite.

The technological behavior of the sepiolite porous ceramic was assessed by simulating, at a laboratory scale, the porous ceramic making process (Fig. 2) and by characterizing the finished products. Firstly, the sepiolite powder and the polyvinyl alcohol aqueous solution were mixed in proportion and then sieved the mixed powder with a 60 meshes screen. Secondly, the mixture was shaped by dry pressing with the molding pressure at 150 MPa for 5 minutes. The compressive strength study was carried out on disc test-pieces with the dimension of 20 mm in diameter and 5 mm thick, while the flexural strength study was conducted on rectangular bars with the dimensions of 40 × 6 × 4 mm. Thirdly, the shaped samples were dried in a drying oven at 80 °C for 5 h to remove moisture in the green body. Fourth, all the samples were heated at a heating rate of 5 °C/min in a programmable box-type resistance furnace at a sintering temperature from 700 °C to 1200 °C in steps of 100 °C for a period of 2 h respectively. Then, the power was turned off and the sintered samples were cooled down to room temperature naturally in the furnace.

### Characterization

The bending strength and the compressive strength of sintered samples were both measured with a microcomputer control electron universal tester (Model 6104) by a three-point bending test with a lower span of 30 mm and crosshead speed of 0.5 mm/min, based on CNS GB/T 1965–1996 and CNS GB/T 1964–1996 respectively.

The thermal behavior of the raw sepiolite was tested by TG-DTA from 30 °C to 1300 °C in ambient conditions at a heating rate of 10 °C/min, with the calcined α-Al_2_O_3_ as a reference substance, using a US TA’s SDT Q-600 analyzer. The crystalline phases present in the sintered samples were analyzed by XRD, scanned from 2θ = 5 to 70°, at a scanning speed of 6°/min, using a Da Vinci type X-ray diffractometer from the Broker AXS (with Cu Kα radiation and Ni filter, λ = 0.154 nm) at 40 kV and 40 mA. The porosity of the sintered samples was investigated by mercury intrusion porosimeter (MIP), using an Auto Pore IV 9500 mercury analyzer from the Micromeritics. A Nova Nano SEM450 scanning electron microscope from the FEI (operating at 1 kV) was used for microstructural examination of samples with backscattered electron images used predominantly.

The shrinkage behavior of green samples was carried out by a horizontal dilatometer (DIL 402). The error of the length sensor is less than 0.1 um, and the force applied on the sample is only 0.2 N to obtain accurate shrinkage data. The temperature is controlled by a thermocouple that is in direct contact with the sample. The initial length of the sample used for the stepwise isothermal dilatometry test (SID) was 40.80 mm. Time (s), temperature (°C), and length shrinkage (mm) data were recorded by a special program automatically. The temperature holding steps were 900°C, 950°C, 1000°C, 1050°C, 1100°C, 1150°C and 1200 °C for 30 min, respectively, and the ramping rate between the isothermal holdings, 10 °C/min.

### Empirical equation and sintering data analysis

The data for the ceramic sintering process can be obtained by measuring the macroscopic volume change of the sample. Meng and Sorensen used the following formula to describe this process^34,35^.

1 $$\frac{{V}_{o}-{V}_{t}}{{V}_{t}-{V}_{f}}={[{\rm{k}}({\rm{T}})({\rm{t}}-{{\rm{t}}}_{{\rm{o}}})]}^{n}$$

where t_o_ is the initial sintering time, V_o_, V_t_ and V_f_ are the starting volume, the volume at time t and the fully dense volume, respectively, and k(T) is the characteristic rate constant. The Arrhenius law is used to calculate the apparent activation energy

2 $${\rm{k}}({\rm{T}})={{\rm{k}}}_{{\rm{o}}}\exp (-{\rm{\Delta }}{\rm{E}}/\mathrm{RT})$$

where k_o_ is the initial rate constant and ∆E is the apparent sintering activation energy. In the case of assuming isotropic sintering, the densification fraction Y can be expressed as follows:

3 $${\rm{Y}}=\frac{{{\rm{V}}}_{{\rm{o}}}-{{\rm{V}}}_{{\rm{t}}}}{{{\rm{V}}}_{{\rm{o}}}-{{\rm{V}}}_{{\rm{f}}}}=\frac{{{\rm{L}}}_{{\rm{o}}}^{3}-{{\rm{L}}}_{{\rm{t}}}^{3}}{{{\rm{L}}}_{{\rm{o}}}^{3}-{{\rm{L}}}_{{\rm{f}}}^{3}}$$

where L_o_, L_t_, L_f_ are the starting length of the green body, the length at time t and the fully dense length respectively. The above formulas are combined to obtain the following results.

4 $$\frac{{{\rm{V}}}_{{\rm{o}}}-{{\rm{V}}}_{{\rm{t}}}}{{{\rm{V}}}_{{\rm{t}}}-{{\rm{V}}}_{{\rm{f}}}}=\frac{{\rm{Y}}}{1-{\rm{Y}}}={[{\rm{k}}({\rm{T}})({\rm{t}}-{{\rm{t}}}_{{\rm{o}}})]}^{{\rm{n}}}$$

A new normalized Makipirtti-Meng rate equation is derived by deriving the two sides of equation (4) and eliminating (t − t_o_).

5 $$\frac{{\rm{dY}}}{{\rm{dt}}}=\mathrm{nk}({\rm{T}}){\rm{Y}}(1-{\rm{Y}}){[\frac{{\rm{Y}}}{1-{\rm{Y}}}]}^{\frac{1}{{\rm{n}}}}$$

Equation 5 is used to deal with the sintering process of porous sepiolite ceramics. Firstly, the value of Y is calculated from the measured values L_o_, L_t_ and L_f_ in SID data. Secondly, the relationship between ln{(dY/dt)[1/Y (1 − Y)]} and ln[(1 − Y)/Y] at each isothermal stage is plotted. If there is a linear relationship between the two, the validity of the equation is proved to be applied to this sintering process. The values of the process parameters n and k(T) can be obtained from the slope and intercept of each straight line segment. Finally, the rate constant k(T) corresponding to different temperatures is plotted to 1/T, and the slope of the straight line is obtained. The values of k_o_ and ∆E are obtained by Arrhenius law.

## Results and Discussion

### Phase transformations

#### Thermal analysis

**Figure 3:**
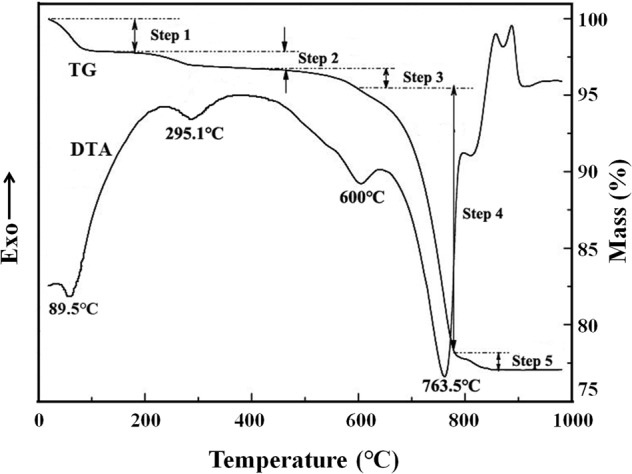
TG and DTA curves of the raw sepiolite.

TG-DTA analysis was employed to study the structural evolution of the raw sepiolite ceramic samples upon heating and the TG and DTA curves were shown in Fig. 3. According to the TG curve, the evolution process could be divided into five stages. Room temperature ~150 °C, the zeolite adsorption water of mass 2.3% was lost, which corresponded to the small endothermic peak of 89.5 °C on the DTA curve. 200 ~ 400 °C, crystal water of mass 1.0% was lost, which corresponded to the endothermic peak of 295.1 °C on the DTA curve. 500 ~ 650 °C, crystal water of mass 1.0% was also lost which corresponded to the obvious endothermic peak of 600 °C on the DTA curve. 700 ~ 800 °C, the mass loss was about 17.5% which was the largest mass loss on the TG curve, correspondingly the maximum endothermic peak appeared at 763.5 °C on the DTA curve. It could be attributed to the decomposition of calcite impurities contained in sepiolite and the formation of sepiolite anhydride after dehydroxylation. Above 800 °C, the DTA curve appeared a small endothermic peak firstly and then two small exothermic peaks with 1.1% mass loss on TG curve. This should be attributed to the formation of new phases in sepiolite.

#### XRD analysis

**Figure 4:**
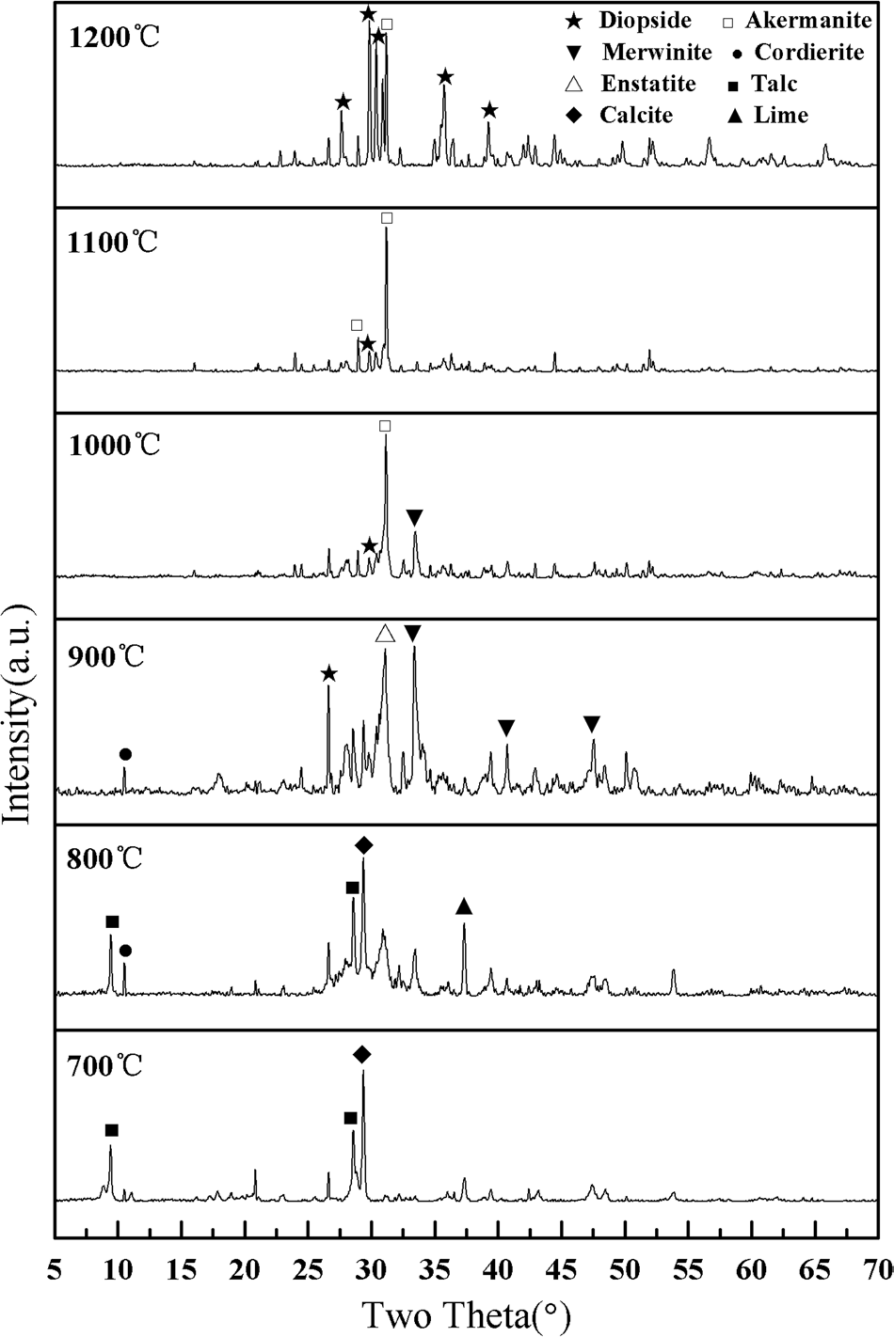
XRD patterns of the porous sepiolite ceramic samples sintered at different temperatures (700 °C to 1200 °C).

The XRD patterns of the sepiolite porous ceramic samples with different sintering temperatures were shown in Fig. 4. It could be seen that the main crystal phase at 700 °C was talc (Mg_3_(OH)_2_Si_4_O_10_) and calcite (CaCO_3_), along with a small amount of lime (CaO). At this point the main crystalline sepiolite phase in the mineral had transformed into the talc phase and the crystalline calcite phase was the main impurity in the mineral. Compared to the 700 °C basically no difference in the sintering products of 800 °C, but the reflection peaks of talc becomes sharper and the emergence of cordierite (Mg_2_Al_4_Si_5_O_18_). When the sintering temperature reached 900 °C, the reflection peaks of talc disappeared, the main crystal phase changed to enstatite (MgSiO_3_) and magnesium calcium silicate (Ca_3_Mg(SiO_4_)_2_) where the enstatite was transformed from talc, also a small amount of cordierite and diopside (CaMgSi_2_O_6_). When the sintering temperature reached 1000 °C, the main phase became akermanite (Ca_2_Mg(Si_2_O_7_)) and diopside, with a small amount of magnesium calcium silicate present. In the case of sintering temperature of 1100 °C, Magnesium calcium silicate was absent from the final products as compared with the tempeture of 1000 °C. At 1200 °C, there were only the akermanite and diopside phases in the samples and the other phases were negligible. The corresponding intensity of the two phase reflections was greatly increased and the reflections became more intense.

### Flexural strength and fracture toughness of ceramic

**Figure 5:**
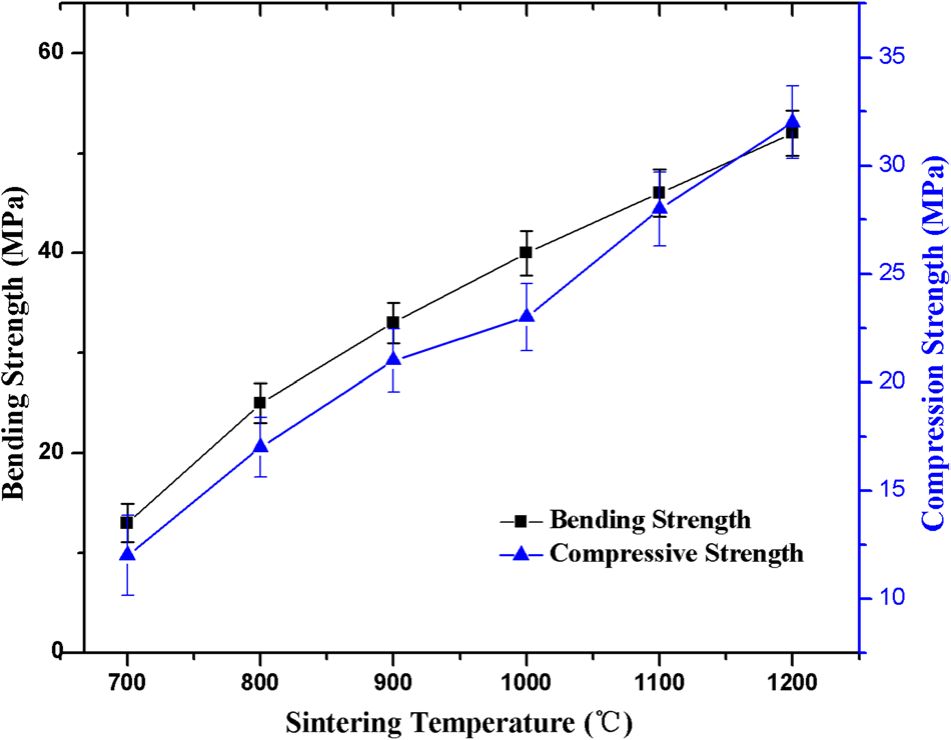
The mechanical performance of sepiolite porous ceramics with increasing tempeture.

 As porous ceramics are used for various applications, it can be inevitably be destroyed by external forces, so its mechanical properties are also very important to its application. The main mechanical properties and robustness of porous ceramics include bending strength and compressive strength. From the trend of Fig. 5, the bending strength and compressive strength of porous ceramics increased with the increase of sintering temperatures. The bending strength ranged from 13 MPa to 52 MPa and reached a maximum at 1200 °C; In a similar trend, the compressive strength ranges from 12 MPa to 32 MPa and also reaches a maximum at 1200 °C.

### Microstructure analysis

#### Mercury test analysis

**Figure 6:**
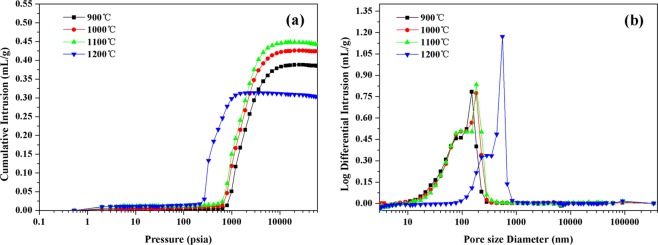
Effect of sintering temperature on the pore structure of porous sepiolite ceramics.

The porosity and pore size distribution of porous sepiolite ceramics were shown in the Fig. 6, in which (a) was the porosity curve and (b) was the pore size distribution curve. From the figure (a), it could be seen that as the pressure increased, the amount of mercury intrusion continued to increase and eventually stabilized at the maximum. With the increasing sintering temperature, the maximum mercury content of the samples increased first and then decreased. That means, with the increase of the sintering temperature, the porosity of the porous sepiolite ceramic increased first and then decreased. From the data in Table 2, the porosity of the porous sepiolite ceramic increased from 51.93% at 900 °C to 55.40% at 1100 °C and then decreased to 46.48% at 1200 °C. When the sintering temperature rose to 1200 °C, some oxides such as calcium oxide and magnesium oxide acted as a sintering aid and caused the liquid phase sintering. This led to the increase of density and decrease of porosity of the porous ceramic.

**Table 2:** The results of porous ceramic mercury injection test.

Sintering Temperature/°C	Sample Quality/g	Median Pore Diameter (Volume)/nm	Actual Mercury Volume/mL/g	Porosity/%	Bulk Density/g/mL	Apparent Density/g/mL
900	0.8439	106.5	0.3848	51.93	1.3498	2.8082
1000	0.4091	122.5	0.4238	54.71	1.2908	2.8499
1100	0.7315	132.0	0.4416	55.40	1.2545	2.8128.
1200	0.4055	513.0	0.3023	46.48	1.5375	2.8729

It could be seen from the Fig. 6(b) that at any sintering temperature, the pore size distribution of the porous ceramic sample showed the unimodal distribution. At 900 °C, the pore size of the ceramic was mainly concentrated in the vicinity of the peaks of 150 nm. At 1200 °C, the pore size of the green body was mainly distributed around 553 nm, that is, the internal pore size reached 0.553 μm. From 1000 °C to 1200 °C, the main reason for the dramatic increase in pore size was the liquid phase sintering. Under the action of the liquid phase sintering, small particles gradually disappeared and larger particles grew up, resulting in the disappearance of small pores or fusion into larger holes^28^.

#### SEM analysis

**Figure 7:**
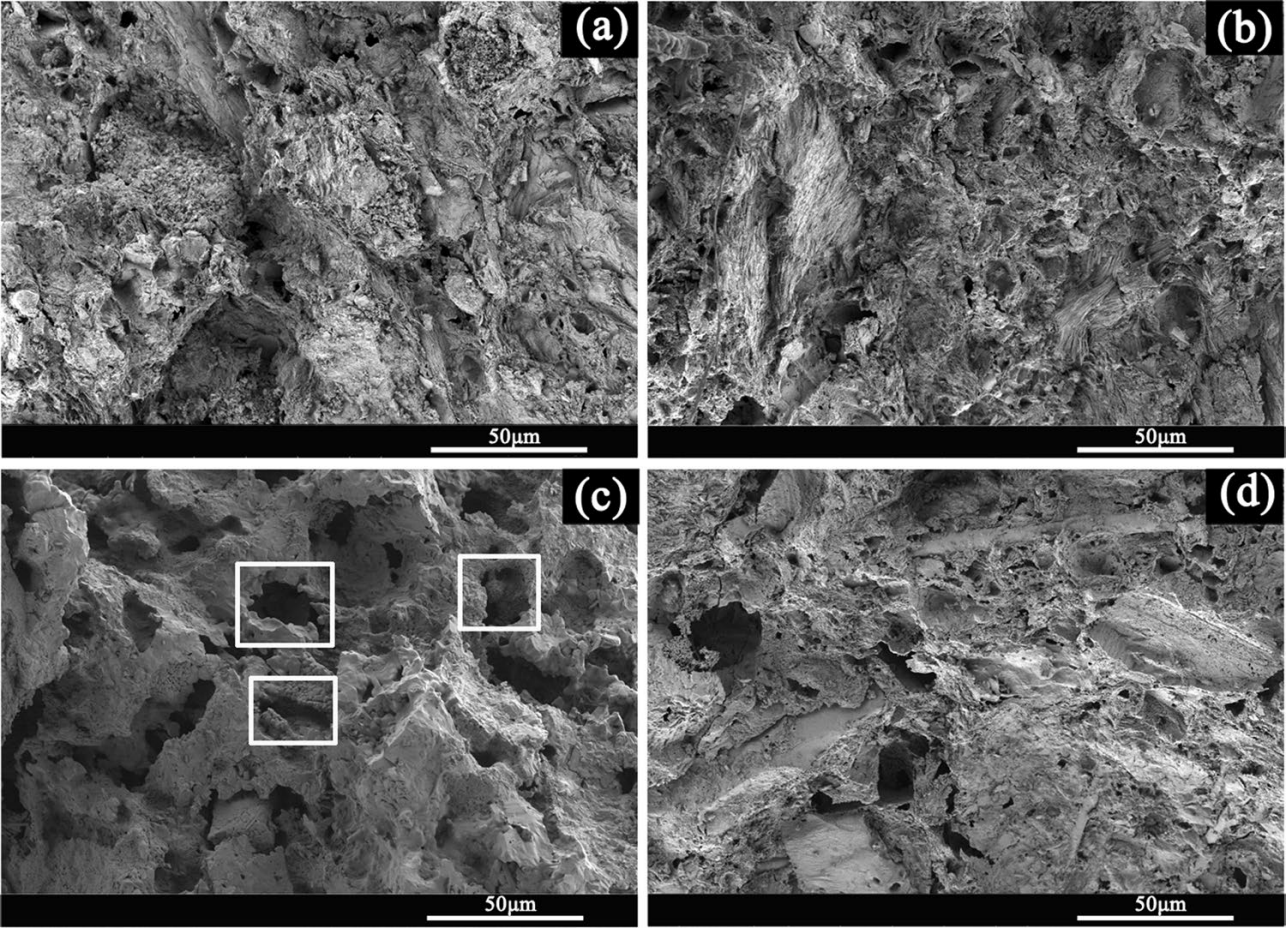
The cross-section SEM images of sepiolite porous ceramics sintering at different temperature. (**a**) 900 °C (**b**) 1000 °C (**c**) 1100 °C (**d**) 1200 °C.

Figure 7 showed the cross-section microstructures of the sepiolite porous ceramic produced at different sintering temperatures. It could be seen from the figure that the microstructure of porous ceramics was mainly composed of the skeleton, which was stacked by sepiolite particles and different sizes of holes. The holes connected to each other and formed the three-dimensional channels. When the sintering temperature was 900 °C, the sepiolite particles combined with each other loosely and there was not obvious sintering phenomenon. Some sepiolite fibers could be observed. When the sintering temperature rose to 1000 °C, the bonding phenomenon between sepiolite particles appeared due to the sintering and the swelling phenomenon of sepiolite fibers was observed. When the sintering temperature reached 1100 °C, it was apparent that the pores inside the sepiolite ceramic increased and the pore size was uniform. At 1200 °C, the pores in the sepiolite ceramic body were significantly reduced and some bigger holes appeared. This was consistent with the mercury pressure test results in Table 2.

There were two main reasons for the formation of pores in the sepiolite ceramics. First, because the fibrous structure of sepiolite is relatively loose, in the process of forming, the accumulation of the fibers between the fibers is not tight, which leads to the formation of holes in the sintering process. And the pore size and porosity in the porous sepiolite ceramics can be regulated and controlled by the addition of other clay minerals. Second, sepiolite contains a large amount of calcite (CaCO_3_) impurities, which are decomposed into carbon dioxide gas in the sintering process to form holes. The gas can’t be removed in time, which leads to the formation of some holes in the body.

### Sintering kinetic data analysis

**Figure 8:**
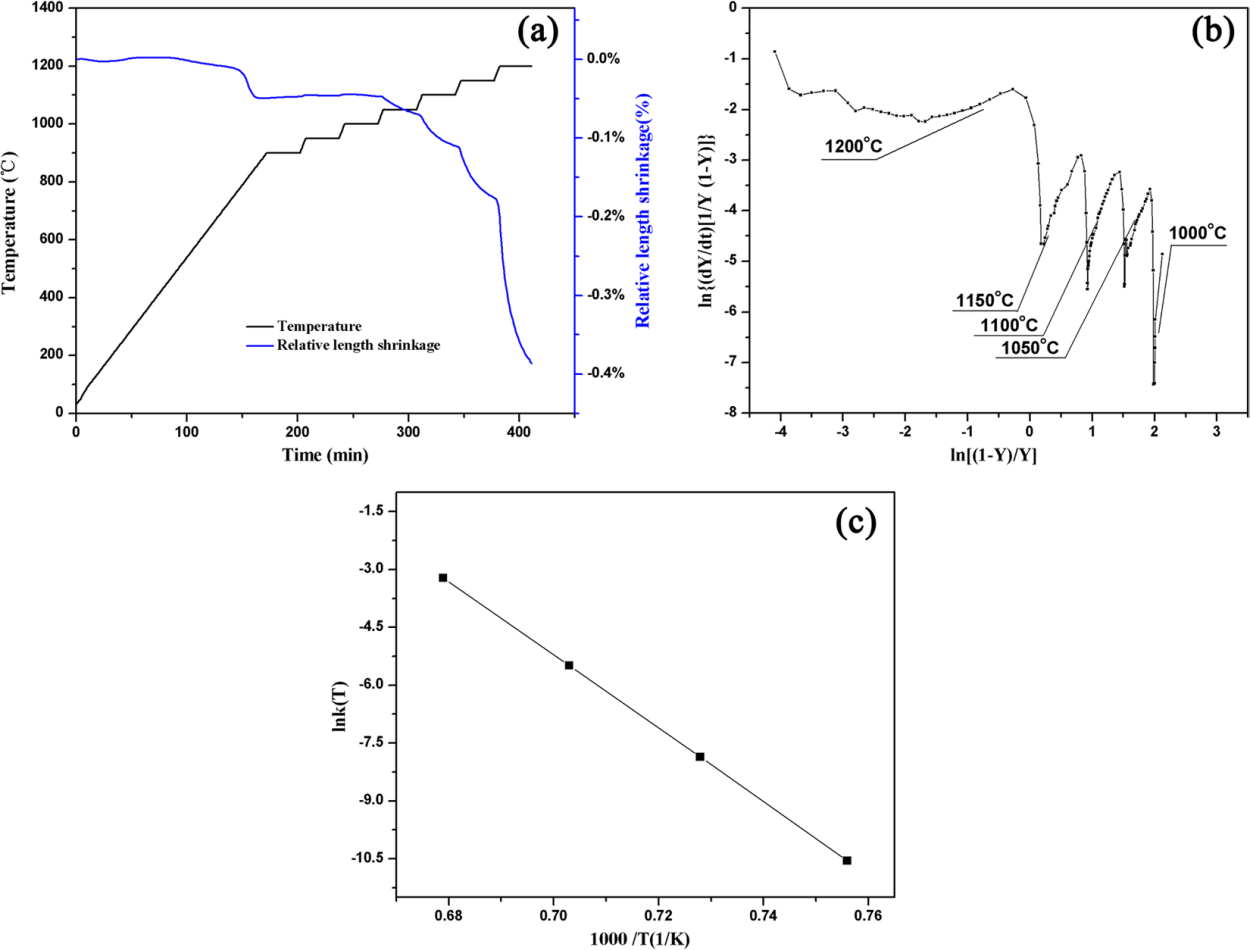
Sintering kinetic (**a**) Stepwise isothermal diatometry shrinkage curve of porous sepiolite ceramics (**b**) Plots of ln{(dY/dt)[1/Y (1 − Y)]} vs ln[(1 − Y)/Y] (**c**) Curve of relationship between 1000/T and ln k(T).

The sample length shrinkage and temperature versus time during the heating process were shown in Fig. 8(a). The formation of Fig. 8(b) curve was that the related data in Fig. 8(a) were converted into the form of ln{(dY/dt)[1/Y (1 − Y)]} to ln[(1 − Y)/Y] according to equation (5). It could be seen from Fig. 8(c) that there was a very good linear relationship between each of the isothermal sections. The slope and intercept of each isothermal forging fitting line were calculated to obtain the values of n and ln[k(T)]. Relevant data was shown in Table 3.

**Table 3:** The results of linear fitting of porous sepiolite ceramics.

Temperature/°C	1000/T(K^−1^)	ln[nk(T)]	1/n	n	lnk(T)	R
1200	0.679	−1.438	0.561	1.781	−3.219	0.996
1150	0.703	−5.142	2.852	0.351	−5.493	0.974
1100	0.728	−7.542	3.114	0.321	−7.863	0.958
1050	0.756	−10.266	3.505	0.285	−10.551	0.986
1000	0.786	−24.903	10.646	0.094	−24.997	0.776

The relationship between ln k(T) and 1000/T obtained from the data in Table 3 was shown in Fig. 8(c). The data point corresponding to 1200 °C was discarded because of the large error after fitting. It could be found that ln k(T) and 1000/T exhibited a linear relationship. In conclusion, the sintering activation energy of porous sepiolite ceramics was measured as 791.42 kJ/mol at 1000 to 1200 °C.

## Conclusions

Sepiolite can be used as a raw material for ceramic green bodies, and can also improve the plasticity and mechanical  properties as a component of a composite. Based on this, the raw sepiolite mineral was used to prepare the porous ceramic and the green body began to sinter at 1000 °C. It was shown that the sintering temperature had a crucial effect on the performance, microstructure and phase composition of the porous sepiolite ceramic. The mechanical properties increased with the increasing sintering temperature and reached the maximum of bending strength of 52 MPa and compressive strength of 32 MPa at 1200 °C. The porosity increased with the sintering temperature until 1100 °C of 55.40% and decreased to 46.48% at 1200 °C. At 1200 °C, the pores in the body followed the unimodal distribution and mainly distributed near the peak of 553 nm. The microstructure of porous ceramics was mainly composed of the skeleton structure formed by the accumulation of sepiolite particles and the calcite impurities in it. When the sintering temperature was higher than 1100 °C, the main crystal phases of the porous ceramics were akermanite and diopside. The apparent activation energy *∆*E value obtained for 1000–1200 °C was 791.42 kJ/mol by means of stepwise isothermal dilatometry.
